# Feeding problems in infancy and diet quality in later childhood: a prospective cohort study

**DOI:** 10.1186/s12937-026-01311-z

**Published:** 2026-05-18

**Authors:** Emma Jankowski, Diane L. Putnick, Akghar Ghassabian, Priscilla K. Clayton, Tzu-Chun Lin, Edwina H. Yeung

**Affiliations:** 1 Epidemiology Branch, Division of Population Health Research, Division of Intramural Research, Eunice Kennedy Shriver National Institute of Child Health and Human Development, NIH, 6710B Rockledge Dr, MSC 7004, Bethesda, MD 20817 USA; 2 Division of Epidemiology, College of Public Health, The Ohio State University, Columbus, OH 43210 USA; 3 Grossman School of Medicine, New York University, New York, NY 10016 USA; 4 Glotech, Inc., 1801 Research Boulevard #605, Rockville, MD 20850 USA

**Keywords:** Children, Feeding problems, Diet quality, Healthy eating

## Abstract

**Background:**

Feeding problems affect many children, yet their connection with diet quality in later childhood is unknown, despite the important role of diet quality on health outcomes, such as obesity.

**Objective:**

To examine feeding problem behaviors and diet quality in later childhood.

**Design:**

The frequency of problematic feeding behaviors (i.e., items related to food refusal, feeding difficulties, and distress) were parent-reported at 18 and 24 months in a birth cohort (2008–2010) from New York state (*n* = 4,989). In this previously validated feeding behaviors scale, parents responded to how often their child engaged in behaviors such as crying/screaming during meals and food refusal, to create an average feeding problems score. Typical daily servings of food items that a child consumed were parent-reported at 30 months, 36 months, 7 years, and 9 years and diet quality using the Youth Healthy Eating Index (YHEI) was calculated, with a higher score indicating better diet quality. Mixed effects analyses calculated mean differences in YHEI per unit increase in feeding problem scores, adjusting for sociodemographic and health-related factors, such as parental education, gestational age, and any breastfeeding duration.

**Results:**

Feeding problem scores at 18 and 24 months were prospectively associated with lower YHEI scores at 30/36 months (18 months: B = -1.10, 95% CI: -1.93, -0.27; 24 months: B = -1.56, 95% CI: -2.33, -0.79). Similar associations were observed at 7/9 years (18 months: B = -1.30, 95% CI: -2.61, 0.00; 24 months: B = -1.65, 95% CI: -2.96, -0.34).

**Conclusions:**

Higher feeding problems in infancy (18 and 24 months) were associated with lower quality diets in childhood, even after children started school. These findings highlight the importance of feeding behaviors to long-term diet quality even with observations as early as 18 months.

**Trial registration:**

NCT03106493 in clinicaltrials.gov (Dates: 07/2008–11/2019, Study Registration Date: 2017–04-10).

**Supplementary Information:**

The online version contains supplementary material available at https://doi.org/10.1186/s12937-026-01311-z.

## Introduction

Feeding problems are a common problem in children, with estimates indicating that more than 25% of children exhibit behaviors aligning with feeding problems [1, 2]. Feeding problems encapsulate a wide spectrum of behaviors, including refusal to eat or picky eating, vomiting or spitting up food, difficulties swallowing and others. Currently, there is little research on how feeding problems in infancy connect to diet quality later in childhood. This is particularly concerning, given that evidence has shown that persistent feeding problems can be associated with impeded growth and undernutrition [3–5], developmental problems [6], and can potentially transform into eating disorders (e.g. anorexia nervosa) in young adulthood [7]. Findings related to feeding problems are especially useful, given they are a common concern and stressor for parents [2, 8].

Research often defines feeding problem behaviors narrowly, focusing only on food refusal, or “picky eating.” There is no uniform or concise definition of “picky eating,” so it may be differently interpreted between cases [9–11]. Nevertheless, a 2018 narrative review of 38 studies found “picky eaters” ate fewer food groups overall, but results on the intake of specific nutrients such as fiber, protein, and micronutrients were inconsistent [3]. Additionally, even when nutrient intake was lower among picky eaters, their intake still fell close to recommended guidelines [3]. The review recommended the use of dietary indices and scales (such as the Youth Healthy Eating Index) to evaluate both diet quality and nutrient intake concurrently, as this provides more easily interpretable information understood by the general population than examining the intake of individual foods or nutrients.

Additionally, most studies on feeding problem behaviors have been cross-sectional in nature, making it hard to identify the long-term impacts of feeding problems on childhood health and nutrition [3]. The longitudinal studies which have examined feeding problems in infancy on later outcomes, including our own work [12, 13], have been in their relation to later developmental outcomes [14], or anthropometry [15–18], which may not be indicative of nutrition or diet quality. For instance, one study observed that all participating girls consumed less than the daily recommended amount of fruits and vegetables, with picky eaters consuming even fewer vegetables than non-picky eaters, but grew normally in weight and had a lower risk of obesity than non-picky eaters [15].

Based on this prior research, this study will fill in critical data gaps by examining behavioral feeding problems present at 18 and 24 months, and how they impact diet quality at 30 and 36 months. Secondarily, we will examine the impact of infant feeding problems on diet quality at 7- and 9-years-old, which is after school initiation, given that school has been associated with changing diet quality [19]. This period is understudied in terms of nutrition [20] and including this time period allows us to examine the long-term impact of infant feeding problems. We hypothesize that, due to the importance of infancy in forming dietary habits, those who are deemed to have more problematic feeding behaviors will have poorer diet quality than their peers. We also hypothesize that this relationship will be continuous, with those exhibiting more feeding problems having a lower diet quality persisting throughout childhood.

## Subjects and methods

### Participants

The Upstate KIDS Study is a population-based birth cohort (2008–2010) of children in 57 counties in New York state, excluding children in New York City. This study was originally designed to evaluate the impact of fertility treatment on child growth and development through the age of 3 years [21]. Information was extracted from birth certificates, and infants who were conceived via fertility treatment and all multiples born between 2008 and 2010 were eligible and invited to participate. Singletons who were not conceived by any fertility treatment were recruited at a 3:1 ratio frequency matched on their region of birth [22]. A total of 5,034 mothers (27.2% of 18,479 approached) and 6,171 children were recruited into the study when children were 4 months old. Co-twins and higher order multiples were excluded from the current analysis, with one child being randomly selected from each mother (*n* = 4,989).

### Ethics

Human subjects approval was obtained from all participating institutions (NYSDOH IRB #07–097; UAlbany #08–179), and informed consent was obtained via parents or legal guardians prior to data collection.

### Procedures

A baseline questionnaire at 4 months assessed demographic factors, and vital records were abstracted for details about the birth and children. Mothers completed questionnaires about their child’s feeding behaviors at 18 and 24 months. They also completed questionnaires about their child’s diet at 30 and 36 months, as well as 7- and 9-years-old.

### Variables

#### Feeding problems

Mothers responded to questions, adapted from a scale used in previous research [4, 5] about how often their children engaged in behaviors indicating feeding problems (*never, rarely, sometimes, often*). This instrument was subsequently validated in cross-cultural research with parents of 6- to 24-month olds [23]. At 18 months, 9 questions were asked about behaviors indicating distress (e.g., “cries/screams during meals”), food refusal (e.g., “push food/spoon away”, “closes mouth when offered food”), and mechanical feeding difficulties (e.g., “can’t chew solid food”, “gags on food”) [12, 13]. At 24 months, the same 9 plus 2 additional questions were asked about refusing a specific meal or type of food (for a total of 11 items), as these behaviors indicate feeding problems at older ages. At each age, the scores from individual items were averaged to create scales, giving a composite score and subscales that indicate the frequency of feeding problems overall and distress, food avoidance, and mechanical problem subscales. Internal consistency (α) reliability ranged from 0.82–0.85 for the total scale and from 0.61–0.80 for the subscales. 

#### Diet quality

Parents reported their children’s intake of food items at 30-months, 36-months, 7-years, and 9-years-old [24]. During toddlerhood (30/36 months), the parents were simply asked to report average total servings the child usually ate of each of a dozen food items and not specifically for a certain duration. Foods included grains (rice, pasta, bread), dairy/yogurt/cheese, potatoes, legumes, vegetables (other than legumes and potatoes), fruits, soy, sweets, meat (beef, poultry, pork), fish/seafood, peanut butter, and eggs. Other behaviors such as frequency of fast food consumption and multi-vitamin use were also requested (Supplementary Material 2).

At 7–9 years, the dietary assessment from the Year 6 Follow-up to the Infant Feeding Practices Study II was used [25]. The 27-items captured intake over the past month. The Youth Healthy Eating Index (YHEI) was calculated from the reported intakes at these 4 time points. The YHEI was adapted from the Healthy Eating Index for use in children and adolescents in order to capture diet quality [26]. This scale combines the reported average daily servings of a variety of foods, including whole grains, vegetables, fruits, dairy, meat, snack foods, and soda/drinks. This is combined with the frequency of multivitamin use, margarine and butter, eating fast food, visible animal fat, eating breakfast, and eating dinner with family to form a quality score (range 0–100), with a higher score indicating a higher diet quality [26]. Servings of whole grains, margarine and butter, and visible animal fat were not collected at 30/36 months, so they were excluded from the YHEI at all timepoints in our analysis (range 0–80), in order to preserve comparability. YHEI was calculated at 30-months, 36-months, 7-years, and 9-years-old. The final score calculation of the YHEI has previously been described elsewhere [27]. We then treated our diet quality outcome measures as repeated measures at the two time points (30 and 36 months for pre-school age and 7 and 9 years for school age diet quality YHEI).

#### Covariates

Mothers’ age and parity (nulliparous vs. multiparous), child plurality (singleton vs. twin), gestational age in weeks, fertility treatment, and sex (male vs. female) were primarily obtained from vital records and supplemented with maternal reports when necessary. Mothers reported their education (as well as the father’s), marital status, race/ethnicity, insurance coverage, and BMI pre-pregnancy. Symptoms of maternal depression were taken at 2 years using the abridged Edinburgh Postnatal Depression Scale [28], and were used as a continuous variable. The child’s food allergies and the duration of any breastfeeding were also reported at each questionnaire. Hospital discharge records and vital records supplemented these maternal reports as available and needed. Food allergies was operationalized as having any food allergy reported from parents of a physician diagnosis on any questionnaire between the exposure and outcome. We derived duration of breastfeeding using surveys collected at 4–5, 8, and 12 months, which indicated whether they were currently breastfeeding, formula feeding, or both. The date the parent reported ending breastfeeding and the final survey date where they indicated breastfeeding were used to determine the total duration of breastfeeding. These durations were then categorized into only formula feeding, breastfeeding for fewer than 6 months, breastfeeding between 6 months and one year, and breastfeeding for longer than one year, regardless of exclusivity of breastfeeding. Maternal/paternal education was dichotomized as less than college vs. college degree or higher. Marital status was coded as married or living as married vs. not. Race/ethnicity was coded as non-Hispanic White vs. other (due to small numbers of non-Hispanic Black (43), non-Hispanic Asian (72), Hispanics (132), and mixed/other race or ethnicity (49)). Insurance coverage was dichotomized as private insurance versus not. Fertility treatment was categorized as no treatment, ovulation induction/intrauterine insemination (OI/IUI) or assisted reproductive technology (ART).

### Statistics

First, we examined 18-month feeding problems and 24-month feeding problems, as separate exposures, to evaluate how early these feeding problems may be tied to later diet quality. We analyzed participants with data on pre-school diet quality at 30- and/or 36-months of age, and then for school-age diet quality at 7 and 9 years separately to see if associations persist before and after school initiation.

All statistical analyses were performed using StataSE 18 and SAS v9.4. To examine the relationship between infant feeding problems and diet quality over time, multi-level mixed effects analysis with a compound symmetry covariance structure and robust error estimates were performed to account for repeated YHEI outcome data from the same participant. We then ran the analysis with three separate models, controlling for covariates identified through existing literature [16, 29–32]: maternal age, parity, plurality, gestational age, child’s sex, maternal marital status, maternal depression, parental education, insurance status, maternal BMI pre-pregnancy, race/ethnicity, and the presence of food allergies in Model 1. As breastfeeding ability could have been due to issues in feeding before capture at 18-months, we first examined the model without its adjustment in case of over-adjustment bias, and then added it to the covariates in Model 2. Model 3 additionally included maternal depression (which was missing in ~ 20.5%) and fertility treatment (to further capture high socioeconomic status [33]). Participants were excluded from the analyses if they were missing any exposure, outcome, or covariate information needed for the model. Two sensitivity analyses were performed. First, children diagnosed with congenital malformations from the New York State Department of Health Congenital Malformations Registry and those with diagnosed intellectual disabilities were excluded. Malformations included chromosomal abnormalities like Down syndrome and structural issues like cleft lip/palate and heart defects, many of which may impact feeding. We additionally excluded children with intellectual disabilities who are also prone to feeding problems [34]. In our second sensitivity analysis, we applied multiple imputation by chained equations (MICE) in STATA for missing outcome, exposure, and covariate information to assess the impact of missingness on the results, creating 50 separate datasets for each of the main (model 2) analyses at 30/36 m and at 7/9 years.

## Results

**Fig. 1:**
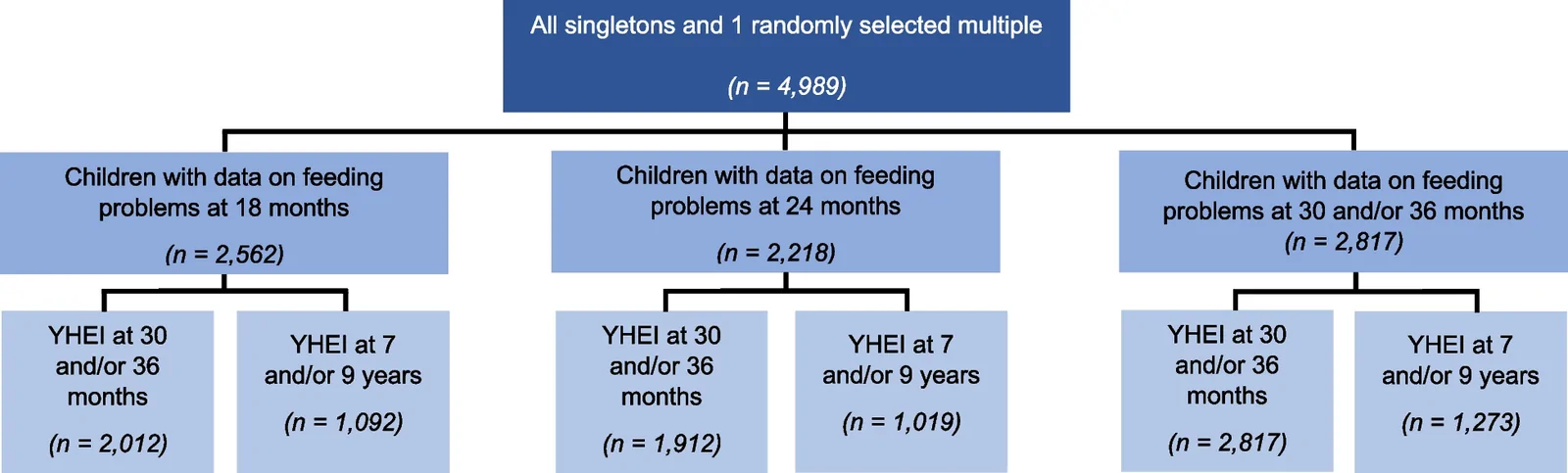
Flow diagram detailing the sample size for each analysis

The sample size for each analysis is presented in Fig. 1; participants were excluded for non-response/item missingness at the exposure and outcome timepoints. Descriptive statistics are presented in Table 1 among those who have data for feeding problems at the first assessment (18 months) and YHEI data at one or more timepoints. Mothers were mostly non-Hispanic white (*n* = 1,793, 85.8%), had a college degree or greater (*n* = 1,316, 63.1%), were married or living as married (*n* = 1,876, 92.2%), and were privately insured (*n* = 1,754, 84.00%). Children were mostly singletons (*n* = 1,716, 82.1%) and not conceived via fertility treatment (*n* = 1,345, 64.4%). Differences among included participants and those excluded are presented in Table S1. Symptoms of maternal depression, child’s sex, and the presence of congenital malformations did not differ between the analytic sample and those excluded; however, the other demographic variables did differ between groups. This is to be expected, as it is well-known that those who are lost to follow-up in longitudinal studies tend to have lower socioeconomic status than those who are not [35, 36]. However, there was no indication of differential responses by the exposures or outcomes (Table S2). Of the 2,089 participants in our analytic sample, 1,175 (56.25%) had completed exposure/outcome data at one exposure timepoint (18 and/or 24 months), one childhood outcome timepoint (30 and/or 36 months), and one later child outcome timepoint (7 and/or 9 years). Many families returned questionnaires inconsistently due to the frequency of being asked every 4–6 months for the first 3 years. When we compared scores of those who responded, even when they were missing either the 18 month questionnaire for feeding problems or the subsequent 30/36 month or 7/9 year questionnaires for YHEI, there were no significant differences in these values between respondents. In other words, those who did not provide YHEI data later on were not more likely to have had children with feeding problems and those who had no feeding problems information were not likely to have had better or worse dietary quality (Table S2).

**Table 1:** Characteristics of the analytic sample

Variable	n (%)/mean (SD)
n	2,089
Maternal Characteristics	
Age, in years (Mean, SD)	31.57 (5.70)
Race/Ethnicity: Non-Hispanic White	1,793 (85.8%)
Nulliparous	997 (48.26%)
College Degree or Greater	1,316 (63.06%)
Married or living as married	1,876 (92.19%)
Maternal pre-pregnancy BMI, in kg/m^2^ (mean, SD)	26.53 (6.43)
Privately Insured	1,754 (84.00%)
Maternal Depression (mean, SD)	2.21 (2.33)
Paternal Characteristics	
College Degree or Greater	1,044 (50.43%)
Child Characteristics	
Gestational Age, in weeks (mean, SD)	38.22 (2.39)
Male	1,079 (51.65%)
Singleton	1,716 (82.14%)
Any food allergy at 36 months	218 (10.44%)
Any food allergy at 9 years	274 (13.12%)
Any congenital malformation	136 (6.51%)
*Breastfeeding Duration*	
formula-feeding only	375 (19.08%)
< 6 months	800 (40.71%)
6- < 12 months	394 (20.05%)
> = 12 months	396 (20.15%)
*Fertility Treatment*	
No fertility treatment	1,345 (64.38%)
OI/IUI	385 (18.43%)
ART	359 (17.19%)

^**^number of missing values: age (0), race/ethnicity (0), parity (23), maternal education (2), marital status (54), pre-pregnancy BMI (3), insurance status (1), maternal depression (428), paternal education (19), gestational age (0), child’s sex (0), plurality (0), food allergy at 36 months (1), food allergy at 9 years (0), malformations (0), breastfeeding duration (124), and infertility treatment (0)

For those who have data for feeding problems at first exposure (18 months) and YHEI data at one or more timepoints (Table 2), the mean feeding problems score at 18 months was 1.14 and at 24 months was 1.00. This means that, on average, infants experienced all 9 feeding problems between rarely (1) and sometimes (2). Despite these low means, 21% of children were rated as experiencing one or more of the feeding problem items often (3) at 18 months and 22% were rated as experiencing one or more of the feeding problem items often at 24 months. For the YHEI, there was a mean of 43.68 at 30 months, 44.03 at 36 months, 53.94 at 7 years, and 49.55 at 9 years of age (Table 2).

**Table 2:** Distribution of feeding problem scores in infancy, and Youth Healthy Eating Index scores in pre-school and school age children

Variable	N*	Mean (SD)	Range
18 Months Feeding Problems	2,089	1.14 (0.46)	0–3
Distress subscale		1.24 (0.57)	
Food avoidance subscale		1.37 (0.62)	
Mechanical subscale		0.81 (0.56)	
24 Months Feeding Problems	1,552	1.00 (0.49)	0–3
Distress subscale		0.94 (0.62)	
Food avoidance subscale		1.27 (0.61)	
Mechanical subscale		0.63 (0.55)	
30-month Healthy Eating Index	1,683	43.68 (9.52)	0–80
36-month Healthy Eating Index	1,673	44.03 (9.12)	0–80
7-year Healthy Eating Index	990	53.94 (9.29)	0–80
9-year Healthy Eating Index	573	49.55 (11.15)	0–80

^*^For consistency, table was limited to children with 18 month feeding problems and at least one HEI (even though models with 24-months are not limited to those with 18-month information)

### Feeding problems and pre-school diet quality (at 30/36 months)

Early feeding problems were prospectively associated with lower diet quality at 30–36 months (Table 3). The unadjusted association between feeding problems at 18-months and the YHEI at 30 and 36 months showed a negative relationship, indicating that increasing early infant feeding problems were associated with a lower diet quality (B = −1.31, 95% CI: −2.16, −0.46, *p* = 0.002). After adjusting for covariates, including separately for breastfeeding, the relationship remained significant and of similar magnitude. Specifically, a one-point increase in feeding problems at 18 months was associated with a 1.10 point decrease in diet quality (95% CI: −1.93, −0.27, *p* = 0.010). Similarly, a one-point increase in feeding problems at 24 months was associated with a 1.56 point decrease in diet quality (95% CI: −2.33, −0.79, *p* < 0.001). Adjustment for covariates impacted the associations between the 3 feeding problems subscales (i.e., mealtime distress, food avoidance, and mechanical problems) with diet quality more than the composite scale results. Nevertheless, higher food avoidance was consistently associated with decreased YHEI scores across all models and time points. The associations with feeding problems remained robust to additional adjustment for maternal depression and fertility treatment (Table S3).

**Table 3:** Mean differences (95% CI) in diet quality scores at 30/36-months by each unit increase in feeding problems score

Exposure	Unadjusted model (95% CI)	p	Model 1* (95% CI)	p	Model 2** (95% CI)	p
18 months	*n* = 2,012		*n* = 1,921		*n* = 1,810	
Feeding Problems	−1.31 (−2.16, −0.46)	0.002	−1.05 (−1.88, −0.22)	0.014	−1.10 (−1.93, −0.27)	0.010
Distress	−0.66 (−1.33, 0.02)	0.056	−0.50 (−1.16, 0.17)	0.141	−0.50 (−1.17, 0.16)	0.138
Food avoidance	−0.95 (−1.58, −0.32)	0.003	−0.99 (−1.62, −0.36)	0.002	−1.05 (−1.67, −0.42)	0.001
Mechanical	−0.80 (−1.51, −0.10)	0.026	−0.39 (−1.08, 0.30)	0.268	−0.40 (−1.09, 0.29)	0.252
24 months	*n* = 1,912		*n* = 1,824		*n* = 1,713	
Feeding Problems	−1.88 (−2.68, −1.09)	< 0.001	−1.73 (−2.50, −0.95)	< 0.001	−1.56 (−2.33, −0.79)	< 0.001
Distress	−0.97 (−1.60, −0.34)	0.003	−0.65 (−1.27, −0.04)	0.035	−0.49 (−1.11, 0.12)	0.114
Food avoidance	−1.71 (−2.34, −1.07)	< 0.001	−1.88 (−2.51, −1.24)	< 0.001	−1.84 (−2.47, −1.21)	< 0.001
Mechanical	−0.79 (−1.50, −0.07)	0.030	−0.36 (−1.06, 0.34)	0.314	−0.14 (−0.84, 0.56)	0.695

^*^Model 1 adjusted for maternal age, maternal race/ethnicity, parity, maternal education, paternal education, maternal marital status, maternal pre-pregnancy BMI, insurance status, gestational age, child’s sex, plurality, and the presence of food allergies

^**^Model 2 adjusted for Model 1 covariates + duration of breastfeeding

### Feeding problems and elementary school diet quality (at 7/9 years)

Early feeding problems were prospectively associated with lower diet quality at 7 and 9 years (Table 4). After adjusting for covariates including breastfeeding, a one-point increase in infant feeding problems at 18-months was associated with a 1.30 point decrease in diet quality during the early elementary school years (95% CI: −2.61, 0.003, *p* = 0.051). Similarly, a one-point increase in feeding problems at 24-months was associated with a 1.65 point decrease in diet quality in fully adjusted models (95% CI: −2.96, −0.34, *p* = 0.014). Adjustment for covariates attenuated the associations between mechanical feeding problems and mealtime distress with diet quality results. Similar to the 30/36-month diet quality models, food avoidance remained significantly associated with diet quality in all models, including additional adjustment for maternal depression and fertility treatment (Table S3).

**Table 4:** Mean differences (95% CI) in diet quality scores at 7/9-years by each unit increase in feeding problems score

Exposure	Unadjusted model (95% CI)	p	Model 1* (95% CI)	p	Model 2** (95% CI)	p
18 months	*n* = 1,092		*n* = 1,049		*n* = 996	
Feeding Problems	−1.90 (−3.26, −0.55)	0.006	−1.42 (−2.74, −0.09)	0.036	−1.30 (−2.61, 0.00)	0.051
Distress	−1.01 (−2.09, 0.08)	0.069	−0.78 (−1.84, 0.28)	0.151	−0.62 (−1.66, 0.41)	0.235
Food avoidance	−1.35 (−2.35, −0.36)	0.008	−1.26 (−2.24, −0.29)	0.011	−1.36 (−2.32, −0.41)	0.005
Mechanical	−1.14 (−2.27, 0.01)	0.050	−0.50 (−1.62, 0.62)	0.382	−0.28 (−1.42, 0.86)	0.628
24 months	*n* = 1,019		*n* = 981		*n* = 937	
Feeding Problems	−2.44 (−3.76, −1.12)	< 0.001	−1.99 (−3.30, −0.67)	0.003	−1.65 (−2.96, −0.34)	0.014
Distress	−1.31 (−2.32, −0.31)	0.010	−0.70 (−1.70, 0.30)	0.171	−0.46 (−1.45, 0.54)	0.367
Food avoidance	−1.90 (−2.95, −0.85)	<0.001	−1.93 (−2.98, −0.88)	< 0.001	−1.79 (−2.82, −0.75)	0.001
Mechanical	−1.47 (−2.69, −0.26)	0.017	−0.80 (−2.03, 0.43)	0.203	−0.41 (−1.64, 0.83)	0.516

^*^ Model 1 adjusted for maternal age, maternal race/ethnicity, parity, maternal education, paternal education, maternal marital status, maternal pre-pregnancy BMI, insurance status, gestational age, child’s sex, plurality, and the presence of food allergies

^**^ Model 2 adjusted for Model 1 covariates + duration of breastfeeding

### Sensitivity analyses

In the sensitivity analysis, excluding those with congenital malformations or intellectual disabilities, the results for preschool (Table S4) and for school age (Table S5) did not materially change in magnitude or interpretation. The results also remained the same in the sensitivity analysis that implemented multiple imputations for the main feeding behavior scores and YHEI as well as any missing covariate information (Tables S6). These results are further evidence that loss to follow-up (or selective non-response) in our sample did not meaningfully impact the results.

## Discussion

This prospective, longitudinal study found that feeding problems in infancy (18 and 24-months) were associated with lower diet quality in childhood, both before (30/36-months) and after (7/9-years) school introduction. This pattern held true even after accounting for demographic variables, important parental characteristics, and historical feeding behaviors (e.g. history of breastfeeding, food allergies). This relationship stood after removing participants with diagnosed congenital malformations and intellectual disabilities, who may be more impacted by feeding problems.

The relationships between dietary quality and feeding problems at 18- and 24-months were largely the same for both time points. Although effect sizes were slightly larger in 24-month exposures than in 18 months, the confidence intervals overlapped. This finding is evidence that diet quality is connected with feeding problems as early as 18 months, and this relationship continues even after school attendance. The subscales had more variation in their relationship with diet quality. Specifically, the mechanical feeding problems and distress subscales were not significantly related to diet quality in any of our fully adjusted models. However, food avoidance, which included items that most clearly captured the concept of “picky eating” were strongly connected with diet quality in all of our fully adjusted models, likely due to it being the most commonly reported feeding problem. This aligns with previous research, which shows that picky eating behaviors remain relatively stable over time, and typically start at early ages [37].

These results are consistent with previous literature, which has linked picky eating to a less diverse diet [38, 39] and less consumption of fruits and vegetables [29, 38, 40]. Additionally, a study of 2,371 children ages 12–48 months using 24-h dietary recall showed that those toddlers considered to be “picky eaters” were less likely to eat a variety of foods, such as vegetables, eggs, and meats [30]. The YHEI utilized in this study takes dietary variety into account in its score, with a lower variety indicating a lower quality diet. Additionally, a variety of studies on picky eating (primarily of school-aged children) have found significantly lower intake of micronutrients among picky eaters compared to their non-picky counterparts [3, 41]. This is consistent with a 2018 narrative review, which included studies with participants of various age ranges, including infants through adolescents, that found that picky eaters tend to consume fewer vegetables, but were inconclusive regarding other food groups, energy, macronutrients, or vitamins and minerals [3]. Our study builds on previous evidence of links between picky eating and overall diet quality by evaluating feeding problems more broadly, as well as prospectively looking at their impact over time. Nevertheless, the overall diet quality among the children was not ideal, in keeping with the general U.S. pediatric population [42].

There are several strengths of this study. To our knowledge, this is the first longitudinal study of the connection between feeding problems in infancy and diet quality in toddlerhood and childhood. Previous studies have been cross-sectional in nature and/or limited to specialized populations [3, 15, 29]. Additionally, our use of a comprehensive feeding problems scale, rather than a narrow and highly subjective definition of “picky eating,” as well as a dietary index (YHEI) allows for a more comprehensive view of the relationship between infant feeding problems and dietary quality in childhood, and has been called for in previous research [3]. Lastly, our study measured feeding problems earlier in infancy than other, similar studies, which only measure as early as 24 months of age [29, 38–40]. Our inclusion of 18 months allows us to see the connection between feeding problems earlier in infancy and diet quality.

There are also several limitations to our research. First, non-response and loss to follow-up decreased our sample over time. However, to account for this, we used repeated outcomes at 30/36 months and 7/9 years. Additionally, this non-response did not appear to bias our measures, as the average feeding problems and diet quality between those missing data and those retained did not significantly differ even if they did not report information at all time points (Table S2). Additionally, our results remained robust by multiple imputations (Table S6). Another limitation is the use of the YHEI, which has not been validated [43]. The original scale included items around whole grain, margarine/butter, and visible animal fat consumption, whereas ours did not. However, the developers of the YHEI found that the addition of margarine/butter and animal fat did not significantly contribute to the variation in the scale [44]. The whole grains category also had the lowest score of the major YHEI components (as whole grains were infrequently consumed by children), meaning these missing categories are unlikely to highly affect the scale [44]. This scale, as well as the feeding problems scale, were parent-reported, which could lead to inflated associations due to shared source variance (e.g., parents who see their children as having feeding problems may report fewer foods or certain foods being eaten). This could potentially lead to an overestimation of this relationship. However, we used composite scores, and previous research has found that parental report of a child’s feeding behaviors tends to be reasonably accurate [45]. Lastly, we were limited by a relatively homogeneous sample. Further research should examine a more diverse pediatric population.

In conclusion, feeding problems in infancy (18 and 24 months) were associated with lower diet quality in later childhood (30/36 months), even after school introduction (7/9 years). Sensitivity analysis and adjustment revealed that this relationship was not primarily driven by congenital malformations/intellectual disabilities or other demographic factors. Future studies should examine the relationship between feeding problems and diet quality in a more diverse sample, both demographically and geographically, where we may see differing results due to unique environmental and social factors including influences of culture on communal rather than individualized portions. This study adds to the previous literature on the topic of feeding problems in infancy by showing evidence of its longer-term impact on diet quality, and should be used to reinforce the importance of the identification and remediation of feeding problems in children.

## Supplementary Information

Supplementary Material 1. Table S1. Characteristics of the analytic sample with 18-month feeding problems measures and YHEI scores (at 30-month, 36-month, 7-year, and/or 9-year) compared to those lost to follow-up. Table S2. Distribution of feeding problem scores in infancy and pre-school age, and Youth Healthy Eating Index scores in pre-school and school aged children compared to those lost to follow-up. Table S3. Mean differences (95% CI) in diet quality scores by each unit increase in feeding problems score, additionally adjusted for fertility treatment and maternal depression. Table S4. Mean differences (95% CI) in diet quality scores at 30/36-months by each unit increase in feeding problems score, excluding children with diagnosed congenital malformations and intellectual disabilities. Table S5. Mean differences (95% CI) in diet quality scores at 7/9-years by each unit increase in feeding problems score, excluding children with diagnosed congenital malformations and intellectual disabilities. Table S6. Mean differences (95% CI) in diet quality scores by each unit increase in feeding problems score with multiple imputations (*n*= 4,989).

Supplementary Material 2. Upstate KIDS dietary questionnaire items for derivation of the YHEI in toddlers (30/36 months).

## Data Availability

Data described in the manuscript will be made available upon reasonable request to the corresponding author [EY].

## Abbreviations

ART: Assisted Reproductive Technology
BMI: Body Mass Index
HEI: Healthy Eating Index
OI/IUI: Ovulation Induction/Intra-uterine Insemination
YHEI: Youth Healthy Eating Index
