# Supplementary material for: Feeding problems in infancy and diet quality in later childhood: a prospective cohort study
Source: Nutr J. 2026 May 18;25:99. doi: 10.1186/s12937-026-01311-z (PMC13573370; doi:10.1186/s12937-026-01311-z)
Supplement: Supplementary file 1 [file 12937_2026_1311_MOESM1_ESM.docx]

**Supplemental Materials**

Table S1. Characteristics of the analytic sample with 18-month feeding problems measures and YHEI scores (at 30-month, 36-month, 7-year, and/or 9-year) compared to those lost to follow-up

| Variable | Frequency (%) included | Frequency (%) NOT included | p-value* |
| --- | --- | --- | --- |
| n | 2,089 | 2,900 |  |
| **Maternal Age** (Mean, SD) | 31.57 (5.70) | 29.62 (6.17) | <0.001 |
| **Race**  Non-Hispanic White  Other | 1,793 (85.83)  296 (14.17) | 2,204 (76.00)  696 (24.00) | <0.001 |
| **Parity**  Nulliparous  Multiparous | 997 (48.26)  1,069 (51.74) | 1,171 (40.58)  1,715 (59.42) | <0.001 |
| **Mother’s Education**  Less than College Degree  College Degree or Greater | 771 (36.94)  1,316 (63.06) | 1,708 (58.92)  1,191 (41.08) | <0.001 |
| **Father’s Education**  Less than College Degree  College Degree or Greater | 1,026 (49.57)  1,044 (50.43) | 1,951 (68.29)  906 (31.71) | <0.001 |
| **Maternal Marital Status**  Married  Not Married | 1,876 (92.19)  159 (7.81) | 2,329 (85.16)  406 (14.84) | <0.001 |
| **Maternal BMI pre-pregnancy** (mean, SD) | 26.53 (6.43) | 27.50 (7.08) | <0.001 |
| **Insurance Status**  Private Insurance  Other | 1,754 (84.00)  334 (16.00) | 1,971 (68.04)  926 (31.96) | <0.001 |
| **Maternal Depression** (mean, SD) | 2.21 (2.33) | 2.36 (2.47) | 0.1723 |
| **Gestational Age** (mean, SD) | 38.22 (2.39) | 37.94 (2.51) | 0.0001 |
| **Sex**  Male  Female | 1,079 (51.65)  1,010 (48.35) | 1,506 (51.93)  1,394 (48.07) | 0.845 |
| **Plurality**  Singleton  Other | 1,716 (82.14)  373 (17.86) | 2,189 (75.48)  711 (24.52) | <0.001 |
| **Any food allergy at 36 months**  Yes  No | 218 (10.44)  1,870 (89.56) | 90 (6.28)  1,344 (93.72) | <0.001 |
| **Any food allergy at 9 years**  Yes  No | 274 (13.12)  1,815 (86.88) | 120 (7.71)  1,437 (92.29) | <0.001 |
| **Any congenital malformation**  Any malformation  No malformation | 136 (6.51)  1,953 (93.49) | 162 (5.59)  2,738 (94.41) | 0.174 |
| **Breastfeeding Duration**  formula-feeding only  <6 months  6-<12 months  >=12 months | 375 (19.08)  800 (40.71)  394 (20.05)  396 (20.15) | 654 (28.62)  1,186 (51.90)  231 (10.11)  214 (9.37) | <0.001 |
| **Fertility Treatment**  No fertility treatment  OI/IUI  ART | 1,345 (64.38)  385 (18.43)  359 (17.19) | 2,187 (75.44)  378 (13.04)  334 (11.52) | <0.001 |

*Comparisons were tested using chi-square for categorical measures and t-tests for continuous measures.

Table S2. Distribution of feeding problem scores in infancy and pre-school age, and Youth Healthy Eating Index scores in pre-school and school aged children compared to those lost to follow-up

|  | n with 18 month feeding problems and at least one HEI | Mean (SD) | n lost-to-follow-up | Mean (SD) | p-value | Range |
| --- | --- | --- | --- | --- | --- | --- |
| **18 Months Feeding Problems**  Distress subscale  Food avoidance subscale  Mechanical subscale | 2,089 | 1.14 (0.46)  1.24 (0.57)  1.37 (0.62)  0.81 (0.56) | 473 | 1.17 (0.46)  1.25 (0.59)  1.41 (0.61)  0.86 (0.55) | 0.1665  0.6852  0.2159  0.0894 | 0-3 |
| **24 Months Feeding Problems**  Distress subscale  Food avoidance subscale  Mechanical subscale | 1,552 | 1.00 (0.49)  0.94 (0.62)  1.27 (0.61)  0.63 (0.55) | 666 | 1.02 (0.49)  0.98 (0.62)  1.27 (0.60)  0.65 (0.54) | 0.4388  0.2212  0.7574  0.5690 | 0-3 |
| **30-month Healthy Eating Index** | 1,683 | 43.68 (9.52) | 621 | 43.34 (9.41) | 0.4427 | 0-80 |
| **36-month Healthy Eating Index** | 1,673 | 44.03 (9.12) | 541 | 43.54 (9.42) | 0.2776 | 0-80 |
| **7-year Healthy Eating Index** | 990 | 53.94 (9.29) | 298 | 52.76 (11.86) | 0.1093 | 0-80 |
| **9-year Healthy Eating Index** | 573 | 49.55 (11.15) | 156 | 50.74 (11.26) | 0.2398 | 0-80 |

Table S3. Mean differences (95% CI) in diet quality scores by each unit increase in feeding problems score, additionally adjusted for fertility treatment and maternal depression

|  | Model 3 Adjusted Beta (95% CI)* | p-value |
| --- | --- | --- |
| **Preschool (30/36 months) YHEI** | | |
| **18-month Feeding Problems (n=1,481)**  Distress subscale  Food avoidance subscale  Mechanical subscale | -0.94 (-1.85, -0.02)  -0.42 (-1.17, 0.33)  -0.94 (-1.64, -0.25)  -0.30 (-1.06, 0.46) | 0.046  0.268  0.008  0.445 |
| **24-month Feeding Problems (n=1,577)**  Distress subscale  Food avoidance subscale  Mechanical subscale | -1.64 (-2.44, -0.85)  -0.54 (-1.18, 0.10)  -1.90 (-2.57, -1.24)  -0.25 (-0.97, 0.47) | <0.001  0.098  <0.001  0.497 |
| **School-age (7/9 years) YHEI** |  |  |
| **18-month Feeding Problems (n = 819)**  Distress subscale  Food avoidance subscale  Mechanical subscale | -1.29 (-2.73, 0.16)  -0.47 (-1.62, 0.69)  -1.63 (-2.68, -0.58)  -0.09 (-1.35, 1.17) | 0.081  0.429  0.002  0.887 |
| **24-month Feeding Problems (n = 863)**  Distress subscale  Food avoidance subscale  Mechanical subscale | -1.42 (-2.78, -0.07)  -0.28 (-1.29, 0.73)  -1.64 (-2.73, -0.56)  -0.47 (-1.75, 0.81) | 0.039  0.589  0.003  0.469 |

* adjusted for maternal age, maternal race/ethnicity, parity, maternal education, paternal education, maternal marital status, maternal pre-pregnancy BMI, insurance status, gestational age, child’s sex, plurality, the presence of food allergies, duration of breastfeeding, fertility treatment, and maternal depressive symptoms

YHEI, youth healthy eating index

Table S4. Mean differences (95% CI) in diet quality scores at 30/36-months by each unit increase in feeding problems score, excluding children with diagnosed congenital malformations and intellectual disabilities

| Exposure | Unadjusted model (95% CI) | p | Model 1*  (95% CI) | p | Model 2**  (95% CI) | p |
| --- | --- | --- | --- | --- | --- | --- |
| **18 months** | n = 1,850 |  | n = 1,767 |  | n = 1,662 |  |
| **Feeding Problems**  Distress  Food avoidance  Mechanical | -1.20 (-2.00, -0.39)  -0.61 (-1.26, 0.04)  -0.93 (-1.53, -0.33)  -0.66 (-1.33, 0.01) | 0.004  0.064  0.002  0.054 | -0.98 (-1.79, -0.17)  -0.51 (-1.16, 0.14)  -0.98 (-1.59, -0.38)  -0.25 (-0.93, 0.42) | 0.018  0.126  0.001  0.461 | -1.00 (-1.82, -0.19)  -0.50 (-1.16, 0.16)  -1.04 (-1.65, -0.44)  -0.23 (-0.91, 0.45) | 0.016  0.138  0.001  0.499 |
| **24 months** | n = 1,757 |  | n = 1,677 |  | n = 1,572 |  |
| **Feeding Problems**  Distress  Food avoidance  Mechanical | -1.79 (-2.54, -1.04)  -0.83 (-1.43, -0.24)  -1.64 (-2.24, -1.04)  -0.80 (-1.48, -0.13) | <0.001  0.006  <0.001  0.020 | -1.69 (-2.45, -0.92)  -0.56 (-1.16, 0.05)  -1.84 (-2.45, -1.22)  -0.43 (-1.12, 0.25) | <0.001  0.071  <0.001  0.214 | -1.55 (-2.32, -0.78)  -0.40 (-1.00, 0.21)  -1.82 (-2.44, -1.21)  -0.26 (-0.94, 0.43) | <0.001  0.201  <0.001  0.467 |

* Model 1 adjusted for maternal age, maternal race/ethnicity, parity, maternal education, paternal education, maternal marital status, maternal pre-pregnancy BMI, insurance status, gestational age, child’s sex, plurality, and the presence of food allergies in a multi-level mixed effects analysis with a compound symmetry covariance structure and robust error estimates

** Model 2 adjusted for Model 1 covariates + duration of breastfeeding

Table S5. Mean differences (95% CI) in diet quality scores at 7/9-years by each unit increase in feeding problems score, excluding children with diagnosed congenital malformations and intellectual disabilities

| Exposure | Unadjusted model (95% CI) | p | Model 1*  (95% CI) | p | Model 2**  (95% CI) | p |
| --- | --- | --- | --- | --- | --- | --- |
| **18 months** | n = 948 |  | n = 908 |  | n = 863 |  |
| **Feeding Problems**  Distress  Food avoidance  Mechanical | -1.82 (-3.02, -0.62)  -0.81 (-1.77, 0.16)  -1.46 (-2.33, -0.59)  -1.06 (-2.09, -0.03) | 0.003  0.100  0.001  0.044 | -1.51 (-2.72, -0.29)  -0.61 (-1.58, 0.36)  -1.46 (-2.34, -0.58)  -0.59 (-1.65, 0.47) | 0.015  0.218  0.001  0.273 | -1.33 (-2.56, -0.10)  -0.41 (-1.40, 0.58)  -1.57 (-2.47, -0.68)  -0.29 (-1.36, 0.78) | 0.034  0.414  0.001  0.591 |
| **24 months** | n = 891 |  | n = 857 |  | n = 819 |  |
| **Feeding Problems**  Distress  Food avoidance  Mechanical  n | -1.96 (-3.12, -0.80)  -0.99 (-1.91, -0.08)  -1.59 (-2.52, -0.66)  -1.12 (-2.18, -0.06) | 0.001  0.034  0.001  0.039 | -1.70 (-2.89, -0.51)  -0.57 (-1.50, 0.36)  -1.68 (-2.63, -0.73)  -0.66 (-1.74, 0.43) | 0.005  0.226  0.001  0.234 | -1.33 (-2.52, -0.14)  -0.30 (-1.23, 0.63)  -1.52 (-2.47, -0.56)  -0.26 (-1.35, 0.84) | 0.029  0.522  0.002  0.647 |

* Model 1 adjusted for maternal age, maternal race/ethnicity, parity, maternal education, paternal education, maternal marital status, maternal pre-pregnancy BMI, insurance status, gestational age, child’s sex, plurality, and the presence of food allergies in a multi-level mixed effects analysis with a compound symmetry covariance structure and robust error estimates

** Model 2 adjusted for Model 1 covariates + duration of breastfeeding

Table S6. Mean differences (95% CI) in diet quality scores by each unit increase in feeding problems score with multiple imputations (n = 4,989)

|  | Unadjusted model (95% CI) | p | Model 1*  (95% CI) | p | Model 2**  (95% CI) | p |
| --- | --- | --- | --- | --- | --- | --- |
| 30/36m YHEI |  |  |  |  |  |  |
| 18m Feeding Problems | -1.38 (-2.29, -0.48) | 0.003 | -1.27 (-2.15, -0.40) | 0.005 | -1.14 (-2.02, -0.27) | 0.011 |
| 24m Feeding Problems | -2.05 (-3.00, -1.10) | <0.001 | -1.72 (-2.68, -0.76) | 0.001 | -1.51 (-2.45, -0.56) | 0.002 |
| 7/9y YHEI |  |  |  |  |  |  |
| 18m Feeding Problems | -2.23 (-3.50, -0.95) | 0.001 | -2.08 (-3.32, -0.85) | 0.001 | -1.91 (-3.11, -0.71) | 0.002 |
| 24m Feeding Problems | -2.52 (-3.78, -1.25) | <0.001 | -2.21 (-3.48, -0.93) | 0.001 | -1.91 (-3.14, -0.68) | 0.003 |

* Model 1 adjusted for maternal age, maternal race/ethnicity, parity, maternal education, paternal education, maternal marital status, maternal pre-pregnancy BMI, insurance status, gestational age, child’s sex, plurality, and the presence of food allergies

** Model 2 adjusted for Model 1 covariates + duration of breastfeeding
