# Supplementary material for: Feeding problems in infancy and diet quality in later childhood: a prospective cohort study
Source: Nutr J. 2026 May 18;25:99. doi: 10.1186/s12937-026-01311-z (PMC13573370; doi:10.1186/s12937-026-01311-z)
Supplement: Supplementary file 2 [file 12937_2026_1311_MOESM2_ESM.docx]

Supplementary Material 2.

Upstate KIDS dietary questionnaire items for derivation of the YHEI in toddlers (30/36 months)

1. Although your child would not necessarily eat all of these foods in one day, on average how many total servings per day of the following foods does your child usually eat?

______ Grains (such as rice, pasta, bread)

______ Dairy milk, yogurt or cheese

______ Potatoes

______ Legumes (such as beans, peas, lentils)

______ Fruits

______ Sweets (such as candy, cakes)

______ Peanut butter (# of tablespoons per day)

______ Eggs (# of eggs per day)

______ Meat (beef, poultry or pork)

______ Fish or seafood

______ Soy food (such as tofu, soy cheese)

______ Vegetables other than potatoes and legumes

3. How often does your child eat from fast food restaurants like McDonald's®, Kentucky Fried Chicken®, Pizza Hut® or Burger King®? (**Select one answer** using an 'X')

[] Daily

[] 5-6 times per week

[] 2-4 times per week

[] Once a week

[] 1-3 times a month

[] Never

5. Which of the following meals does your child usually eat each day? (**Mark all that apply** using an 'X')

[] Breakfast	[] Lunch	[] Dinner

16. For each of the beverages listed below, please tell us how many 8 ounce cups (approximately one sippy cup) your child currently drinks each day.

Beverage          Write in the number of 8oz cups drunk each day

Water	_____________

Soda or pop	_____________

Juice	_____________

Milk	_____________

19. How many days per week on average does your toddler take multivitamins? (**Select one answer using an 'X'**)

[] None

[] 1-2 times per week

[] 3-4 times per week

[] 5-7 times per week
